# Plasmon-Enhanced Fluorescence of EGFP on Short-Range Ordered Ag Nanohole Arrays

**DOI:** 10.3390/nano10122563

**Published:** 2020-12-20

**Authors:** Vladimir E. Bochenkov, Ekaterina M. Lobanova, Aleksander M. Shakhov, Artyom A. Astafiev, Alexey M. Bogdanov, Vadim A. Timoshenko, Anastasia V. Bochenkova

**Affiliations:** 1 Department of Chemistry, Lomonosov Moscow State University, 119991 Moscow, Russia; katerinla95@gmail.com (E.M.L.); astafiev.artyom@gmail.com (A.A.A.); vat2b2@gmail.com (V.A.T.); 2 N.N. Semenov Federal Research Center for Chemical Physics of RAS, 119991 Moscow, Russia; physics2007@yandex.ru; 3 Shemiakin-Ovchinnikov Institute of Bioorganic Chemistry of RAS, 117997 Moscow, Russia; noobissat@ya.ru

**Keywords:** surface plasmon resonance, nanohole arrays, plasmon-enhanced fluorescence, colloidal lithography, Enhanced Green Fluorescent Protein

## Abstract

Fluorescence of organic molecules can be enhanced by plasmonic nanostructures through coupling to their locally amplified electromagnetic field, resulting in higher brightness and better photostability of fluorophores, which is particularly important for bioimaging applications involving fluorescent proteins as genetically encoded biomarkers. Here, we show that a hybrid bionanosystem comprised of a monolayer of Enhanced Green Fluorescent Protein (EGFP) covalently linked to optically thin Ag films with short-range ordered nanohole arrays can exhibit up to 6-fold increased brightness. The largest enhancement factor is observed for nanohole arrays with a propagating surface plasmon mode, tuned to overlap with both excitation and emission of EGFP. The fluorescence lifetime measurements in combination with FDTD simulations provide in-depth insight into the origin of the fluorescence enhancement, showing that the effect is due to the local amplification of the optical field near the edges of the nanoholes. Our results pave the way to improving the photophysical properties of hybrid bionanosystems based on fluorescent proteins at the interface with easily fabricated and tunable plasmonic nanostructures.

## 1. Introduction

Plasmonic nanostructures can focus light from far field sources into tiny subwavelength-scale volumes, leading to amplification of a local electric field [1]. This has many interesting implications, especially when molecules are present in such hot spots, including surface-enhanced spectroscopies [2,3,4], super-resolution imaging [5], ultrasensitive (bio)sensing [6], strong plasmon-exciton coupling [7,8], etc. For instance, dye molecules, placed in the vicinity of a plasmonic nanostructure, can be coupled to this enhanced electric field, resulting in higher excitation and radiative decay rates, increased quantum yields and a larger number of photons emitted before photobleaching. This phenomenon is sometimes referred to as surface-enhanced fluorescence, metal-enhanced fluorescence or plasmon-enhanced fluorescence (PEF) [9,10,11,12].

Recently, extensive development of fluorescent proteins (FPs) has revolutionized cell biology by allowing researchers to label proteins of interest through genetic encoding [13,14]. However, typical FPs possess lower brightness and poorer photostability compared to those of organic dyes [15]. The pioneering work of Lakowicz et al. has demonstrated the possibility of overcoming these issues by PEF. A stronger emission intensity and improved photostability of Green Fluorescent Protein (GFP), physically adsorbed on substrates with pre-deposited silver island films, have been reported [16]. Later, single FP molecules coupled to gold nanorods were studied using super-resolution microscopy [17]. It has been shown that spectral overlap between an emission band of the protein with a specific plasmon mode is important for improved fluorescence.

The choice of a plasmonic nanostructure to be used as a PEF platform depends on a particular problem. For a number of biomedical applications, nanopatterned plasmonic substrates with controlled optical properties and with a dense and uniform distribution of scattering centers are highly desirable. This includes living cell imaging [18] and implantable light sources [19]. Ideally, such systems should be produced by a simple, low-cost and large-area fabrication technique.

Among various plasmonic systems that can be used for fluorescence enhancement are the arrays of nanosized apertures in thin metallic films, or nanohole arrays (NHAs). These plasmonic systems support two types of surface plasmons, propagating surface plasmons (PSPs) and localized surface plasmons (LSPs), which can couple to molecular emitters. Several groups studied fluorescence enhanced by square periodic arrays of nanoholes in metal films, showing that nanoholes in such systems act as a 2D grating, enabling efficient coupling of light to surface plasmons [20,21,22]. A resonance wavelength in such systems can be tuned by changing periodicity as well as the diameter of the holes.

In contrast to periodic NHAs, perforated metal films with a short-range ordered (SRO) arrangement of the holes, fabricated via self-assembly-based sparse colloidal lithography [23,24], do not possess periodicity. Nonetheless, due to a self-limiting nature of sequential adsorption of charged nanospheres, occurring during a mask formation step, they build a low-density layer with rather similar distances between neighboring particles. This results in the formation of NHAs with perturbed hexagonal lattices after metal deposition and lift-off. The diameter of the spheres affects both the hole diameter and the inter-hole spacing, allowing one to tune LSP and PSP resonances of such metasurfaces. The relatively simple large-area fabrication technique combined with good tunability of optical properties makes SRO NHAs in thin plasmonic films an attractive platform for various applications, including (bio)sensing [25,26,27,28], displays [28,29], and even light-induced heating [30]. Recently, SRO nanoholes in optically thin Al films have been utilized to enhance fluorescence of a dibenzoylmethanatoboron difluoride dye dispersed in a polymer matrix [31]. It has been found that an overlap of the NHA extinction peak with fluorophore excitation plays an important role in fluorescence enhancement, suggesting that the effect is due to the increase of the fluorophore’s excitation rate. However, the role of the spontaneous emission in such systems is not clear yet. Furthermore, the microscopic distribution of the hotspots with the highest fluorescence enhancement factor remains unknown.

Here, we study the mechanism of the fluorescence enhancement by SRO nanohole arrays using the Enhanced Green Fluorescent Protein (EGFP), covalently immobilized on perforated optically thin Ag films. By using a combination of spectroscopic studies, lifetime measurements, and FDTD simulations, we show that the overlap of the broad NHA extinction peak with fluorescence and emission of the fluorophore is crucial to the improved photophysical properties of the hybrid nanosystem. The fluorescence enhancement is observed together with reduced radiative lifetimes, revealing the existence of efficient energy transfer between EGFP and the plasmonic nanostructure, which acts as a nanoantenna for light in- and outcoupling in these hybrid bionanosystems. We find the fluorophore positions, that would lead to the strongest fluorescence enhancement. These results pave the way to directed fabrication of plasmonic metasurfaces for biomolecular research, for example, to study surface adsorption of living cells, as well as to explore the structure and the functioning of their membranes.

## 2. Materials and Methods

### 2.1. Materials

We used aqueous dispersions (8 wt%) of sulfate latex particles with an average particle diameter of 120 nm, obtained from Thermo Fisher Scientific (Waltham, MA, USA). PDDA (poly-diallydimethylammoniumchloride) (MW 200,000–350,000), PSS (poly-sodium 4-styrenesulfonate) (MW 70,000), GLYMO ((3-glycidyloxypropyl)-trimethoxysilane) and metallic silver (purity ≥ 99.99%) were purchased from Sigma-Aldrich (St. Louis, MO, USA). Round borosilicate glass slides with thickness 0.2 mm obtained from Menzel-Gläser, Braunschweig, Germany (*d* = 24 mm) were used as substrates. Deionized water with resistivity of 18.2 MΩ was used for substrate cleaning and preparation of aqueous solutions.

### 2.2. Fabrication of SRO Nanohole Arrays

Optically thin perforated Ag films were fabricated using sparse colloidal lithography. In this approach, latex sulfate nanospheres, which were electrostatically adsorbed on a polyelectrolyte-coated substrate, acted a lithographic mask upon vapor deposition of silver [23,24].

The details of the fabrication procedure have been reported elsewhere [23]. Briefly, glass coverslips were cleaned by ultrasonication in acetone (10 min), ethanol (10 min) and MilliQ water (10 min) followed by UV/Ozone oxidation (30 min). Three polyelectrolyte layers were sequentially adsorbed from aqueous solutions in the following order: PDDA (2 wt%), PSS (2 wt%) and PDDA (2 wt%). The samples were rinsed with MilliQ water and dried by compressed air after the deposition of each polymer layer. Sulfate latex particles were self-assembled on the positively charged surface upon deposition from 0.2 wt% aqueous dispersion for 2 min. The samples were then rinsed with MilliQ water for 1 min to remove the non-adsorbed nanospheres and quickly dried by compressed air. Due to negative charges of the sulfate residues at neutral pH, the nanospheres repel each other during deposition. Thus, the process is self-limiting and the adsorbed particles are separated by approximately the same distance. This ensures reproducible results when all experimental parameters are well controlled.

The glass slides with assembled colloidal masks were oxidized by UV/Ozone for 3 min to remove the polyelectrolyte layer and to improve adhesion of metals, which were deposited by thermal evaporation in a PVD system. First, an adhesion layer of Cr (2 nm) was deposited, followed by Ag (15–25 nm) deposition. Then the samples were taken out to air and the nanospheres were removed by tape stripping the particles using a 90 μm blue PVC tape (Semiconductor Production Systems). The size of the holes and their spatial arrangement correspond to those of the colloidal particles that were adsorbed on the surface. A typical SEM image of the two-dimensional SRO nanohole array fabricated using sparse colloidal lithography is shown in the Supplementary Material (Figure S1).

### 2.3. Protein Expression and Purification

EGFP was cloned into the pQE30 vector (Qiagen, Hilden, Germany) with an N-terminal 6His tag, expressed in *E. coli* XL1 Blue strain (Invitrogen, Carlsbad, CA, USA) and purified using TALON metal-affinity resin (Clontech, Mountain View, CA, USA). Imidazole-eluted protein was then desalted (transferred into PBS buffer) using Ultracel 10K centrifugal filters (Amicon, Billerica, MA, USA).

### 2.4. Immobilization of EGFP

Surface immobilization of EGFP on silver NHAs was carried out using GLYMO as a binding agent. First, the samples were additionally treated by UV/Ozone for 30 s to remove the polyelectrolyte layers and to facilitate surface oxidation of silver. Then the samples were placed in a desiccator above a small beaker containing 0.2 mL of GLYMO and evacuated down to residual pressure of about 0.01 mbar. After one hour, the samples were taken out to air and placed into an oven for another hour at temperature of 120 ${}^{{}^{\circ}}$C. The formation of a hydrophobic layer on the surface was controlled by the increased water contact angle from <10${}^{{}^{\circ}}$ to $60^{{}^{\circ}}$. Both perforated and non-perforated Ag films as well as clean glass slides are activated for the protein binding using this procedure.

The protein binding step was carried out right before the fluorescence studies. The covalent binding of EGFP was achieved via the reaction of the GLYMO epoxy groups with one of the protein’s surface amine groups. For each sample, a 20 μL droplet of 0.25 M EGFP solution in phosphate buffered saline (PBS) at pH = 7.4 was placed on top of the GLYMO-modified surface and covered by a microscope slide to prevent water evaporation. After 20 min, the top cover slide was removed and the sample was rinsed by PBS to remove unbound protein molecules. Then, a cover slide was placed on top of the surface with immobilized EGFP molecules with a small amount of the PBS buffer, and the fluorescence measurements were performed. Control samples with EGFP immobilized on bare glass were produced by the same procedure.

### 2.5. Lifetime Measurements and Spectroscopy

Extinction spectra of silver nanohole arrays were registered by a UV-vis-NIR spectrometer (V-770, Jasco, Tokyo, Japan) in air and in MilliQ water. Fluorescence emission spectra of surface-immobilized EGFP were recorded using a custom built optical setup. Femtosecond laser pulses with an 80 MHz repetition rate, 950 nm central wavelength, and energy up to 25 nJ were generated by a Titanium-Sapphire oscillator (Tsunami, Spectra-Physics, Santa Clara, CA, USA). Frequency-doubled pulses at 475 nm were focused by a 40× 0.75NA objective lens (UPlanFLN, Olympus, Shinjuku, Japan) on the sample’s bottom surface. The top surface of the sample with a monolayer of EGFP was covered with PBS and protected from drying by a glass coverslip. The sample was continuously shifted in the XY plane using an automatic scanning system to prevent EGFP bleaching and to average a fluorescence signal across the sample’s surface. The mean laser power, controlled by a polarizing attenuator, was kept at 300 nW for all the samples. The EGFP fluorescence, passed back through the same objective lens and filtered with a 500 nm long-pass filter, was directed at the input of Acton SP300i monochromator with two separate outputs. The PI-MAX 2 CCD camera (Princeton Instruments, Trenton, NJ, USA) at the first output records fluorescence emission spectra. The photomultiplier tube of the time-correlated single photon counting system SPC-730 (Becker & Hickl GmbH, Berlin, Germany) at the second output detects a fluorescence decay kinetics in the range of 510–530 nm. Fluorescence decay kinetics data were processed using Origin 2019 (OriginLab, Northampton, MA, USA) and were fitted using the DecayFit v.1.4 (FluorTools, http://www.fluortools.com) software. Polarization anisotropy of the emitted light was not performed.

### 2.6. Simulations

A commercial-grade 3D electromagnetic simulator, FDTD Solutions (Ansys, Lumerical Inc., Vancouver, BC, Canada), was used to calculate extinction spectra of silver nanohole arrays as well as fluorescence enhancement of EGFP. The model system was represented by a metal film with thickness in a range of 18–38 nm and with 100 nm apertures in it, arranged in a hexagonal 2D lattice with a center-to-center distance of 200 nm. The rims of the holes are rounded to avoid nonphysically high field enhancement near the sharp edges. A mesh size, not larger than 2 nm × 2 nm × 2 nm, was used for the metal region in all simulations. The dielectric function of silver was represented by a numerical fit to the experimental data [32]. Aqueous solution and glass substrate regions were represented by constant refractive index values of *n* = 1.33 and *n* = 1.52, respectively. A periodic hole arrangement was used to simulate the optical properties of SRO nanohole arrays, as it allows to apply periodic boundary conditions in the plane of the film. This substantially reduces the size of the system, yet provides good agreement with experimental results [27,31].

Extinction (1-T) spectra and field enhancement near the NHA surface were simulated using a plane-wave source, which emits light in a spectral range of 400–700 nm. The amplified electric field was recorded near the nanostructure at $\lambda_{exc}$ = 475 nm, corresponding to laser excitation wavelength. These data were used to calculate plasmon-induced enhancement of the EGFP excitation rate.

Fluorescence simulations are carried out using an oscillating dipole source, emitting light at λ = 510 nm, which corresponds to the emission maximum of EGFP. Two sets of monitors were used to calculate the power emitted by the dipole and the power, transmitted to the far field (taking into account losses in the Ag nanostructure). This allows one to calculate relative radiative and non-radiative decay rate constants, a Purcell factor and quantum efficiency.

## 3. Results and Discussion

### 3.1. Fluorescence Enhancement

**Figure 1:**
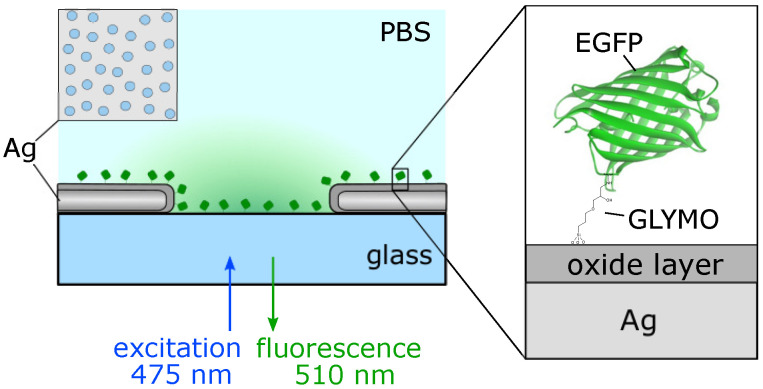
Simplified schematic of the experiment showing the short-range ordered (SRO) nanoholes in the optically thin silver film with immobilized Enhanced Green Fluorescent Protein (EGFP) and the illumination configuration.

A schematic of the EGFP–AgNHA hybrid system and fluorescence measurements is shown in Figure 1. Laser light with λ=475 nm is focused on the sample’s surface with the perforated silver film and the monolayer of the protein molecules. The light passes through the optically thin NHA and excites the S${}_{0}$–S${}_{1}$ electron transition in EGFP. Emitted photons are collected from the same side of the sample.

**Figure 2:**
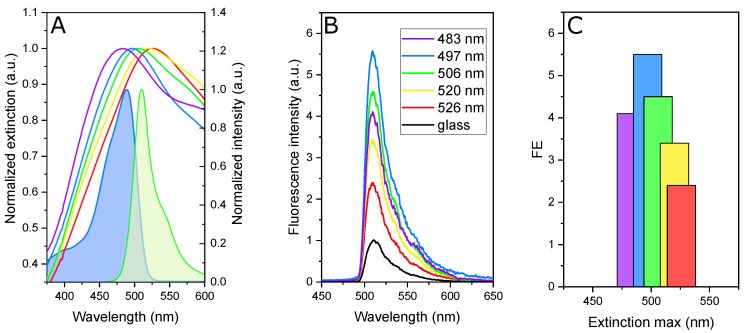
EGFP fluorescence enhancement by the Ag nanohole arrays. (**A**) Normalized extinction spectra of various Ag nanohole arrays (NHAs) (colored lines) and normalized absorption (blue) and emission (green) of EGFP (filled area). (**B**) Relative emission intensity of EGFP immobilized on glass (black) and on AgNHAs (colored lines). The legend indicates the SPR peak positions. (**C**) Fluorescence enhancement factors. The same color code is used in all panels.

The 120 nm AgNHAs are characterized by extinction measurements in water prior to EGFP immobilization. The spectra are presented in Figure 2A. Due to variations in thickness of the Ag layer, the samples possess a surface plasmon resonance (SPR) peak at different wavelengths in a range of 483–526 nm. A large width of the peaks ensures a significant spectral overlap of the SPR band with the EGFP absorption and emission.

To compare fluorescence intensities (*I*) of various samples, we relate them to the emission of the EGFP monolayer immobilized on glass at $\lambda_{max}$ = 510 nm ($I_{510}^{glass}$). Additionally, we take into account light attenuation by the metal layers of various thickness, thus the intensities are also divided by the sample transmittance at excitation wavelength $\lambda_{exc}$ = 475 nm ($T_{475}$). The resulting spectra are presented in Figure 2B. As it can be seen, the EGFP molecules immobilized on 120 nm NHAs demonstrate approximately six times higher fluorescence intensity compared to those attached to glass.

Since the shape of the EGFP emission spectra are not changed due to the presence of a plasmon, a fluorescence enhancement (*FE*) factor can be determined as the ratio between the fluorescence intensity in the hybrid nanosystem at $\lambda_{max}$ = 510 nm and that on the glass (we assume that the amount of EGFP immobilized on glass and NHAs is the same). This relation can be written as follows:

(1) $$FE=\frac{I_{510}}{I_{510}^{glass}T_{475}}$$

*FE* equals to 1 for EGFP linked to the glass surface (no enhancement) and it reaches a highest value of 5.5 in the presence of AgNHA. We note that the position of the maximum of the AgNHA SPR peak plays a crucial role in fluorescence enhancement, as it follows from Figure 2C. The enhancement factor is the largest for NHA with the SPR maximum at 497 nm, and it quickly drops when SPR is shifted off this wavelength.

### 3.2. Fluorescence Decay

**Figure 3:**
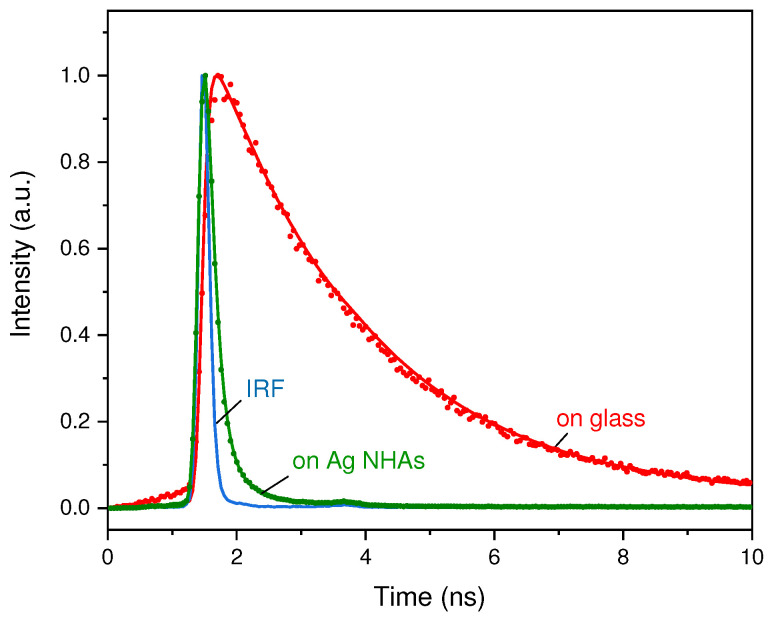
Fluorescence decay kinetics of EGFP immobilized on glass (red dots) and AgNHA (green dots) with the corresponding fits (red and green lines, respectively). Shown is also the instrument response function (blue line).

To get insight into the origin of the EGFP fluorescence enhancement on AgNHAs, we also study the excited-state decay kinetics. The fluorescence lifetime is found to be strongly affected by the presence of NHAs. The normalized fluorescence decay curves obtained for EGFP immobilized on glass and AgNHAs are presented in Figure 3 together with the corresponding fits and the instrument response function (IRF). The fluorescence of EGFP on glass demonstrates a single-exponential decay with a lifetime of $\tau_{0}=2.35\pm 0.1$ ns, which is the same as in the solution. For the AgNHA-bound EGFP, the decay kinetics becomes two-exponential. The first and the fastest component has a characteristic lifetime of $\tau_{1}=0.13\pm 0.01$ ns with a fraction of $\alpha_{1}=97\pm 1\%$. The second component has a lifetime of $\tau_{2}=0.7\pm 0.1$ ns.

The observed two-exponential fluorescence decay suggests the existence of two deexcitation channels for EGFP linked to AgNHA. It is reasonable to suggest that the two different excited state decay rates stem from the protein molecules immobilized directly on the metal and from those linked to the glass in the metal holes. However, there is no direct experimental evidence for this, which can help to identify these processes. Furthermore, for practical applications it is important to know a spatial distribution of hot spots with the highest fluorescence enhancement. To answer these questions we carry out numerical simulations using the FDTD method.

### 3.3. Simulation Results

**Figure 4:**
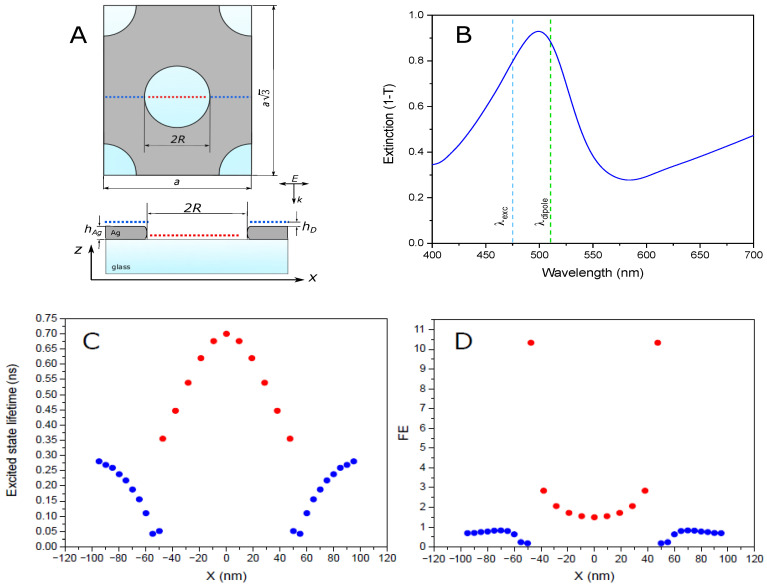
Simulation of the EGFP fluorescence enhancement near periodic 100 nm AgNHA. (**A**) Top and section views of the simulated periodic box; the blue and red dots indicate the positions of a dipole source during the scan (the same color code is used in panels **B**,**C**) Extinction (1-T) spectrum of Ag NHA (blue line). The excitation and oscillating dipole emission wavelengths are shown by the blue and green dashed lines, respectively. (**C**) Excited state lifetime. (**D**) Fluorescence enhancement factor.

The simulated model system is presented in Figure 4A. The red/blue dotted line across the hole demonstrates the positions, which are used to probe various local parameters affecting the fluorescence. The height of these positions above the metal (and glass—inside the hole) is 8 nm.

The calculated extinction spectrum of AgNHA together with the excitation ($\lambda_{exc}$) and EGFP emission ($\lambda_{dipole}$) wavelengths are presented in Figure 4B. The extinction maximum of the simulated spectrum is located at λ≈500 nm, which is close to that observed for the experimental system.

In general, there are two main factors that lead to the plasmon-enhanced fluorescence. The first one is the local amplification of the optical field resulting in the increased fluorophore excitation rate, and the second one is the increased quantum efficiency of the process because of the faster emission near the metal surface (the Purcell effect) [9,33,34]:

(2) $$FE=\frac{{\left|E\right|}^{2}}{|E^{0}{|}^{2}}\frac{\varphi }{\varphi^{0}}=\frac{{\left|E\right|}^{2}}{|E_{0}{|}^{2}}\frac{\tau }{\tau^{0}}\frac{k_{fl}}{k_{fl}^{0}}$$

Here, *FE* is the fluorescence enhancement factor, *E*, φ, τ and $k_{fl}$ are the electric field, the fluorescence quantum yield, the excited-state lifetime, and the radiative rate constant, respectively; the upper index “0” denotes the corresponding values in the absence of a metal.

The calculated variations of the local field enhancement ${\left|E\right|}^{2}/{\left|E^{0}\right|}^{2}$ across the aperture upon illumination at $\lambda_{exc}$ = 475 nm are presented in Figure S2. According to the simulation results, the hot spots with the highest field are localized near the edges of the hole, with the field enhancement exceeding a value of 20. The amplified field quickly drops towards the hole center.

To rationalize the reduced overall fluorescence lifetime observed in the experiment, we calculate τ at different spots across the aperture using the following relation (the full derivation can be found in the Supporting Information):

(3) $$\tau =\frac{\tau^{0}}{\varphi^{0}\left(\frac{k_{fl}}{k_{fl}^{0}}+\frac{k_{loss}}{k_{fl}^{0}}-1\right)+1}$$

The fluorescence quantum yield, $\varphi^{0}$ = 0.6 [35], and the excited state lifetime, $\tau^{0}$ = 2.35 ns, measured in this work, are the intrinsic properties of EGFP; whereas the normalized decay rate constants, $k_{fl}/k_{fl}^{0}$ and $k_{loss}/k_{fl}^{0}$, are calculated using the FDTD method. The results are presented in Figure 4C. It can be seen that the excited-state lifetime of EGFP linked to silver is about 0.3 ns at a distance of 50 nm from the hole, and it drops down to 0.05 ns at the hole’s edge. Inside the hole, the lifetime increases with a distance from the metal and reaches a value of 0.7 ns in the center. The averaged τ values for the points located near the metal (blue) and those in the middle of the hole (red) are well consistent with the two experimentally observed lifetimes, $\tau_{1}=0.13\pm 0.01$ ns and $\tau_{2}=0.7\pm 0.1$ ns. Thus, we attribute them to the two groups of the EGFP molecules immobilized on the metal and on the glass inside the apertures, respectively.

We calculate the metal-induced enhancement of the spontaneous emission rate (k${}_{fl}$/k${}_{fl}^{0}$) as the ratio between the power irradiated by the dipole with and without the metal nanostructure [36]. The results presented in Figure S2 demonstrate that the Purcell factor of almost two orders of magnitude is observed near the top rim of the hole; however, most of the emitted power gets absorbed by silver, and the resulting EGFP fluorescence quantum yield is below 0.1 for the protein molecules directly linked to the metal film. The losses are smaller for EGFP inside the holes, with a quantum yield reaching 0.55 in the center, which is close to that found in solution ($\varphi^{0}=0.6$).

The FE profile is mostly defined by the local electric field, as demonstrated in Figure 4D (see also Figure S2). The strongest enhancement is observed near the bottom edge of the hole. The difference between FE near the top and the bottom surface of the metal film is due to a larger refractive index of the substrate (*n* = 1.52) compared to that of the solution (*n* = 1.33), resulting in asymmetry of the enhanced field distribution along the Z axis. We conclude that the fluorescence enhancement can be even further increased by reducing the substrate effect, for example, by elevating NHA. This has been previously shown to improve refractive index sensitivity of an Au NHA optical sensor [27].

As discussed above, we observe two distinct lifetimes, which can be attributed to the EGFP molecules, immobilized on the metal surface and in the apertures. Since the efficiency of energy transfer and hence the fluorescence lifetime depends on a distance to the metal, it is reasonable to expect that the shortest lifetime corresponds to the protein molecules immobilized on Ag. For such molecules, the estimated distance between the chromophore and the metal is of the order of 2–7 nm, including the thickness of the silver oxide layer, the spacer lengths and the distance from the edge of the β-barrel to the chromophore. The largest lifetime is obtained for the molecules, immobilized on the glass surface in the nanoholes. Within each nanohole, these molecules have different lifetimes, depending on their distance to the nanohole’s wall. However, due to a small fraction (only 2–3%) of the signal coming from these molecules, the experimental decay kinetics can be sufficiently well fitted by a two-exponential decay function, also providing an estimate of EGFP molecules located in the nanoholes. The fraction of these molecules is consistent with the area fraction covered by the holes (about 10%, according to SEM).

Our results show that an average 6-fold fluorescence enhancement can be observed for fluorescent proteins, immobilized on perforated optically thin silver films. SPRs of such films can be varied by changing their thickness and diameters of the holes. This makes it possible to efficiently couple NHAs with various fluorescent proteins. We also reveal the importance of spectral overlap of both excitation and emission of the chromophore with an SPR band of NHAs. This is due to the fact that both excitation and emission rates of fluorescent proteins are sensitive to the presence of NHAs and can be enhanced when the corresponding wavelengths match the NHA SPR band. By combining the experiments with the FDTD simulations, we analyze the mechanism of fluorescence enhancement, which is fully supported by the experimental results. This provides a strategy for the rational design of hybrid bionanosystems with the improved photophysical properties for applications in bioimaging and biosensors.

## 4. Conclusions

By combining experimental and theoretical studies, we show that short-range ordered nanohole arrays in optically thin silver films, which can easily be fabricated on centimeter-scale supports via colloidal lithography, can be used as efficient fluorescence-enhancing substrates. We demonstrate that hybrid bionanostructures, comprised of Enhanced Green Fluorescent Proteins immobilized on perforated silver films with 120 nm apertures exhibit up to 6-fold amplified average fluorescence intensity. For the first time, we show that the strongest emission on SRO NHAs is obtained from the molecules, localized in the holes near the metal walls. This is due to the asymmetric distribution of the electric field caused by the glass substrate having a higher refractive index. By using fluorescence lifetime measurements in combination with FDTD simulations we reveal the detailed mechanism of the EGFP fluorescence enhancement.

Our findings suggest that the fluorescence enhancement factor in such systems can be further increased by reducing the substrate effect via uncovering the part of the amplified electric field otherwise buried in the substrate. High tunability and low fabrication cost of SRO NHAs combined with the newly acquired knowledge of the fluorescence enhancement mechanism can greatly facilitate the application of such systems in bioimaging, for example, for visualizing contact points of surface-adsorbed cells labeled by fluorescent proteins in membranes.

## Supplementary Materials

The following are available online at https://www.mdpi.com/2079-4991/10/12/2563/s1, Figure S1: Typical SEM image of two-dimensional nanohole array fabricated using sparse colloidal lithography with 120 nm latex nanospheres, Figure S2: Simulation of the EGFP fluorescence enhancement near 100 nm AgNHA, Derivation of the relation for a fluorescence lifetime change.
