# Supplementary material for: Plasmon-Enhanced Fluorescence of EGFP on Short-Range Ordered Ag Nanohole Arrays
Source: Nanomaterials (Basel). 2020 Dec 20;10(12):2563. doi: 10.3390/nano10122563 (PMC7767041; doi:10.3390/nano10122563)
Supplement: Supplementary file 1 [file nanomaterials-10-02563-s001.pdf]

Supplementary Materials

Plasmon-Enhanced Fluorescence of EGFP on Short-Range Ordered Ag Nanohole Arrays

Vladimir E. Bochenkov [1,*], Ekaterina M. Lobanova [1], Aleksander M. Shakhov [2], Artyom A. Astafiev [1,2], Alexey M. Bogdanov [3], Vadim A. Timoshenko [1] and Anastasia V. Bochenkova [1,*]

[1] Department of Chemistry, Lomonosov Moscow State University, 119991 Moscow, Russia; katerinla95@gmail.com (E.M.L.); astafiev.artyom@gmail.com (A.A.A.); vat2b2@gmail.com (V.A.T.)

[2] N.N. Semenov Federal Research Center for Chemical Physics of RAS, 119991 Moscow, Russia; physics2007@yandex.ru

[3] Shemiakin-Ovchinnikov Institute of Bioorganic Chemistry of RAS, 117997 Moscow, Russia; noobissat@ya.ru

* Correspondence: boch@kinet.chem.msu.ru (V.E.B.); bochenkova@phys.chem.msu.ru (A.V.B.)

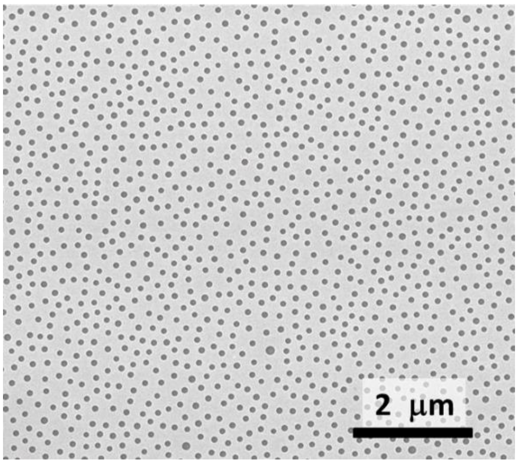

Figure S1. Typical SEM image of two-dimensional nanohole array fabricated using sparse colloidal lithography with 120 nm latex nanospheres.

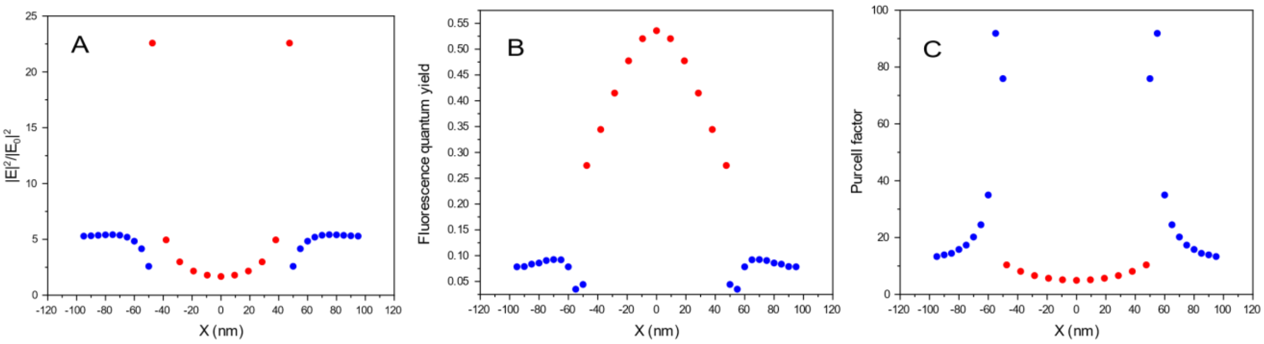

Figure S2. Simulation of the EGFP fluorescence enhancement near 100 nm AgNHA: **A**) electric field enhancement $|E|^2/|E_0|^2$; **B**) Fluorescence quantum yield; **C**) Purcell factor.

Derivation of the relation for a fluorescence lifetime change

The excited state lifetime τ of the fluorophore is defined by the rate constants of radiative k_{fl} and non-radiative k_{nr} relaxation processes:

$$\tau^0 = \frac{1}{k_{fl}^0 + k_{nr}^0} \quad (1)$$

where index "0" denotes processes in the absence of metal.

The quantum yield φ is then defined by:

$$\varphi^0 = \frac{k_{fl}^0}{k_{fl}^0 + k_{nr}^0} \quad (2)$$

In the presence of metal, the radiative rate constant can be changed due to a Purcell effect, and a new non-radiative energy transfer process with the rate constant k_{loss}, can take place, therefore the relations (1) and (2) will be transformed into

$$\tau = \frac{1}{k_{fl} + k_{nr}^0 + k_{loss}} \quad (3)$$

and

$$\varphi = \frac{k_{fl}}{k_{fl} + k_{nr}^0 + k_{loss}} \quad (4)$$

The relative change of the excited state lifetime can be found by dividing (1) by (3):

$$\frac{\tau^0}{\tau} = \varphi^0 \left(\frac{k_{fl}}{k_{fl}^0} + \frac{k_{loss}}{k_{fl}^0} - 1 \right) + 1 \quad (5)$$

Similarly, one can obtain the relation for the quantum yield:

$$\varphi = \varphi^0 \frac{k_{fl}}{k_{fl}^0} \frac{\tau}{\tau^0} \quad (6)$$
